# A de novo nonsense mutation in the tyrosine kinase lyn in a patient with an early onset autoinflammatory phenotype

**DOI:** 10.1186/1546-0096-12-S1-O25

**Published:** 2014-09-17

**Authors:** Adriana Almeida De Jesus, Gina Montealegre, Yin Liu, Bernadette Marrero, Hyesun Kuehn, Katherine Calvo, Sergio Rosenzweig, Thomas Fleisher, Richard Chyi-Chia Lee, April Brundidge, Dawn Chapelle, Yan Huang, Stephen Brooks, Susan Moir, Eric Meffre, Melinda Merchant, Zuoming Deng, Raphaela Goldbach-Mansky

**Affiliations:** 1 National Institute of Arthritis Musculoskeletal and Skin diseases (NIAMS), Bethesda, USA; 2 Department of Laboratory Medicine, Bethesda, USA; 3 National Cancer Institute, Bethesda, USA; 4 National Institute of Allergy and Infectious Diseases, National Institutes of Health, Bethesda, USA; 5 Yale University, New Haven, USA

## Introduction

Lyn kinase is a Src-family tyrosine kinase expressed by hematopoietic and non-hematopoietic cell types. It functions as an activactor and an inhibitor of signaling pathways. Phosphorylation of a tyrosine residue at position 508 keeps the molecule in an inactive form. Lyn^up/up^ mice have a gain-of-function mutation generated at the tyrosine position 508 (Y508F) and present with severe anemia, autoimmune glomerulonephritis and positive ANA. Using whole exome sequencing (WES), we identified a pediatric patient with a nonsense de novo mutation in lyn kinase presenting with an early-onset autoinflammatory phenotype.

## Objectives

To describe the successful characterization by WES of a patient with a de novo mutation in LYN, a gene not previously associated with human disease.

## Methods

Genomic DNA was extracted from total blood of the patient and his parents and WES was performed using human exome capture Agilent V4 enrichment kit and Illumina HiSeq2000 platform.

## Results

A 3 year-old Caucasian male born at 31 weeks’ gestation presented with hydrops fetalis, requiring intra-utero blood transfusion. Post partum he developed hepatosplenomegaly, purpuric skin rash, periorbital erythema and testicular swelling. Laboratory exams showed increased C-reactive protein (CRP) anemia, severe thrombocytopenia and increased liver enzymes. At 9 months of age a splenectomy was performed for refractory thrombocytopenia. Post splenectomy, he developed leukocytosis, thrombocytosis and anemia persisted. A liver biopsy showed periportal lymphocytic infiltrate and vanishing bile duct disease. He had vasculitis on a skin biopsy and circulating autoantibodies (positive ANA, anti-Sm, anti-SSA, anti-phospholipids and anti-mitochondrial antibodies). Despite some improvement after initiation of prednisone and IVIG infusions, prednisone doses could not be weaned. We identified a *de novo* mutation in a candidate gene (*LYN*) by WES. The mutation was confirmed as *de novo* by Sanger sequencing. The change from cytosine to guanine (c.1524C>G) leads to a premature stop-codon at the tyrosine position (Y508*) with loss of the phosphorylation site. B lymphocytes showed a constitutive phosphorylation of lyn and B cell phenotyping showed a significantly diminished frequency of immature B cell populations in peripheral blood and in bone marrow. B cell activation was reduced upon IgM stimulation in comparison with healthy controls. The genetic defect identified in this patient led us to initiate treatment with a tyrosine kinase inhibitor (dasatinib). After 6 months of treatment, clinical improvement was observed with lower frequency of disease flares, resolution of anemia, improvement of leukocytosis, thrombocytosis and liver enzymes. A decrease of prednisone dose to physiologic levels and discontinuation of IVIG infusions were also achieved.

## Conclusion

A lyn kinase nonsense mutation was detected in a patient with a severe early-onset autoinflammatory phenotype. Treatment with dasatinib was shown to be promising in the patient described.

## Disclosure of interest

None declared.

